# Relationship between pruritus and sleep in participants with primary biliary cholangitis in the Phase 2b GLIMMER trial

**DOI:** 10.1186/s41687-024-00722-y

**Published:** 2024-06-12

**Authors:** Robyn von Maltzahn, Marlyn J. Mayo, Helen T. Smith, April Thompson, Sugato Das, Andrea Ribeiro de Souza, Edoardo Lisi, Cynthia Levy, Megan M. McLaughlin, David Jones

**Affiliations:** 1 GSK, London, UK; 2 University of Texas Southwestern Medical Center, Dallas, TX USA; 3 GSK, Research Triangle Park, NC USA; 4 GSK, Hyderabad, India; 5 GSK, Madrid, Spain; 6 Schiff Center for Liver Diseases, Division of Digestive Health and Liver Diseases, University of Miami, Miami, FL USA; 7 GSK, Collegeville, PA USA; 8 Newcastle University, Newcastle, UK

**Keywords:** Primary biliary cholangitis, PBC, Sleep interference, Cholestatic pruritus, IBAT inhibitor, Health-related quality of life

## Abstract

**Background:**

Cholestatic pruritus and fatigue are debilitating conditions associated with primary biliary cholangitis (PBC) and can significantly impact patients’ quality of life. Pruritus in PBC often worsens at night and patients frequently report sleep disturbance, which contributes to cognitive symptoms and fatigue. Linerixibat is an ileal bile acid transporter inhibitor in clinical development for the treatment of pruritus associated with PBC and was recently assessed versus placebo in the Phase 2b GLIMMER trial. This post-hoc analysis assesses the relationship between pruritus severity and sleep disturbance in participants of GLIMMER regardless of treatment group.

**Methods:**

GLIMMER (NCT02966834), a multicenter, double-blind, randomized, placebo-controlled trial, recruited 147 patients with PBC and moderate-to-severe pruritus. Following 4 weeks single-blind placebo, patients (randomized 3:1) received linerixibat or placebo for 12 weeks (to Week 16). Participants graded their itch (twice daily) and its interference with sleep (once daily) in an electronic diary using a 0–10 numerical rating scale (NRS). Weekly and monthly itch scores were calculated as the mean of the worst daily itch score over the respective time period. At study visits, participants completed the 5-D itch scale and the PBC-40 quality of life questionnaire, both of which contain an item specific to itch-related sleep disturbance. The impact of pruritus on sleep was assessed post hoc through correlations between the changes in NRS, 5-D itch, and PBC-40.

**Results:**

Strong correlations were found between change from baseline in weekly itch and sleep NRS scores (r = 0.88 [95% confidence interval (CI): 0.83; 0.91]) at the end of treatment (Week 16), as well as in monthly itch and sleep NRS scores (r = 0.84 [95% CI: 0.80; 0.87]). Patients with improved weekly pruritus score severity category demonstrated reduced perceived sleep interference on average. Itch responders (≥2-point improvement in weekly itch score from baseline) displayed larger improvements in weekly sleep NRS score, 5-D itch, and PBC-40 sleep items, than itch non-responders (<2-point improvement).

**Conclusions:**

A strong correlation exists between changes in pruritus severity and sleep interference in patients with PBC; pruritus reduction could generate concomitant improvement in sleep.

**Supplementary Information:**

The online version contains supplementary material available at 10.1186/s41687-024-00722-y.

## Abstract (plain language summary)

Patients with primary biliary cholangitis (PBC) often experience debilitating itching, which can be worse at night. In the GLIMMER study of linerixibat, patients with PBC and itch also had sleep interference. When the severity of itching was reduced, there was a corresponding improvement in sleep. Reduction in itch is likely to have a beneficial impact on quality of life in patients with PBC.

**Supplementary Information:**

The online version contains supplementary material available at 10.1186/s41687-024-00722-y.

## Background

Primary biliary cholangitis (PBC) is a rare, chronic, autoimmune, cholestatic liver disease that can lead to end-stage liver disease and necessitate liver transplantation [1]. Cholestatic pruritus (referred to by patients as itch) is commonly seen in PBC [2], affecting up to 81% of patients at some point during their disease course [2–5]. It can impair daily activities, adversely impact quality of life, contribute to fatigue, and lead to depression and, in extreme cases, suicidal ideation [5–8]. Patients with PBC who experience pruritus report worse fatigue scores than those who do not report pruritus [9, 10].

Pruritus in PBC tends to increase throughout the day and is often worse at night [11, 12]. When assessed at five timepoints (from wake time to sleep time), patients with PBC (n = 74) showed an increase in perceived pruritus over the course of a day, with the peak reported at bedtime [12].

In a study of patient perspectives by the PBCers organization, 65% of respondents with PBC and pruritus (n = 164) reported that their pruritus was worse at night [11]. One of the most prominent effects of pruritus is sleep disturbance, with 74% of patients in the same study reporting that pruritus interferes with their sleep [11]. Up to 20% of patients with PBC in the UK-PBC cohort reported that pruritus *frequently or always* affected their sleep [2]. In addition, patients with PBC who have higher pruritus scores have longer sleep latency, earlier wake times and increased daytime somnolence [12, 13], which in turn correlates with fatigue [13].

In a series of qualitative interviews with 20 patients with PBC and at least moderate pruritus, the symptoms reported by patients as having most impact fell into the subdomains ‘changes in daily performance’, ‘emotional functioning’, and ‘sleep difficulties caused by itching or other symptoms’ [14]. Thus, it is clear, sleep disturbance has a negative impact on the daily lives of patients with PBC.

An effective treatment for cholestatic pruritus in PBC may influence patient wellbeing by improving quality of life, which is affected by both pruritus and its impact on sleep. Current treatments for PBC, such as the first-line US Food and Drug Administration (FDA)-approved ursodeoxycholic acid (UDCA), have not been shown to improve either pruritus or sleep [15, 16]. Obeticholic acid, a conditionally approved adjunctive therapy to UDCA or monotherapy for those unable to tolerate UDCA for the treatment of PBC, has been associated with a greater incidence of severe pruritus in clinical studies than placebo, a reaction listed as a warning and precaution within the US and EU prescribing information [17–19]. Guideline-recommended anti-pruritic strategies for PBC include the bile acid-binding resin cholestyramine and off-label therapies including rifampicin, naltrexone, and sertraline [20]. Off-label fibrates have also been used to manage cholestatic itch [21]. Present therapies are associated with tolerability issues, have insufficient clinical evidence, and/or are often ineffective in ameliorating pruritus in PBC [20].

Recent studies have assessed the potential of ileal bile acid transporter (IBAT) inhibitors for the treatment of pruritus in some pediatric cholestatic liver diseases [22]. The accumulation of systemic bile acids in cholestasis is hypothesized to have a causal role in cholestatic pruritus, and the impact of reduced bile acids has supported this theory [20, 23]. IBAT inhibitors block the enterohepatic circulation of bile acids, thereby reducing systemic bile acid levels and increasing fecal bile acid excretion [24, 25]. The IBAT inhibitor maralixibat was recently approved in Europe and the USA for the treatment of cholestatic pruritus in pediatric patients with Alagille syndrome [26, 27], but did not significantly improve pruritus compared with placebo in a Phase 2 study in patients with PBC [28]. Odevixibat, another IBAT inhibitor approved by the FDA and the European Medicines Agency for the treatment of pruritus in pediatric familial intrahepatic cholestasis, has demonstrated improvements in sleep [29–33].

Linerixibat, a minimally absorbed small molecule IBAT inhibitor, showed significant improvements in pruritus and reductions in nighttime sleep interference due to pruritus versus placebo in a Phase 2a study of patients with PBC when taken at 45 mg twice daily (BID) for 3 days followed by 90 mg BID for 11 days [24]. The Phase 2b GLIMMER study (NCT02966834) is the largest randomized investigational study of patients with PBC with cholestatic pruritus to date. In this study, linerixibat dose-dependently ameliorated pruritus in the per protocol population of patients with moderate-to-severe pruritus over 12 weeks of treatment [34], indicating the potential of linerixibat as a future treatment option. Mean daily sleep scores improved for all treatment groups, including placebo, and there was a high concordance between improvements in pruritus and sleep. This study aims to examine the relationship between pruritus severity and sleep disturbance in patients with PBC by utilizing the entire population of the GLIMMER study. We therefore conducted a post-hoc analysis of the GLIMMER study across all patients to assess the relationship between the incidence of pruritus, pruritus severity, and the impact of pruritus on sleep interference in patients with moderate-to-severe cholestatic pruritus.

## Methods

### Study design and patients

Details of the Phase 2b GLIMMER study design have been published elsewhere [34]. In brief, 147 patients, aged 18–80 years, with confirmed PBC and cholestatic pruritus were included in the study. Patients with moderate-to-severe pruritus, defined as ≥ 4 on a 0 (no itching) to 10 (worst imaginable itching) numerical rating scale (NRS), were eligible to be enrolled in the study (patient disposition has previously been reported) [34]. The study was composed of four periods: initial, main, final, and follow-up. Baseline was at the end of the initial study period, a 4-week single-blind placebo phase (Day 1 to Week 4) during which patient baseline symptoms were recorded in an electronic diary (eDiary). Patients who had completed ≥ 10 of the 14 daily eDiary entries in the final 7 days of the initial study period and had an NRS worst daily itch score of ≥ 3 on at least 5 of the previous 7 days were eligible for randomization to the main study period. Patients were randomized (3:1) to receive double-blind treatment of one of five linerixibat regimens (20 mg once daily [QD], 90 mg QD, 180 mg QD, 40 mg BID, and 90 mg BID) or placebo for 12 weeks (Weeks 4–16) [34]. Patients then entered the final study period where they received single-blind placebo for a further 4 weeks (Weeks 16–20) to evaluate symptom return and safety. The final study period was followed by 4-weeks follow-up (Weeks 20–24) in which symptoms were assessed via telephone.

The study was performed in accordance with the Declaration of Helsinki, International Conference on Technical Requirements for Registration of Pharmaceuticals for Human Use Good Clinical Practice, and applicable country-specific requirements. The study protocol, any amendments, informed consent, and other information that required pre-approval were reviewed and approved by a national, regional, or investigational center ethics committee or institutional review board. Written informed consent was obtained from all patients before participation.

### Measurement of pruritus, pruritus impact on sleep, and quality of life

Patients were asked to record the severity of their pruritus using a 0 (no itch) to 10 (worst imaginable itching) NRS twice daily (morning and evening) in an eDiary throughout the initial, main, and final study periods (Day 1 to Week 20) [2, 35]. Daily, weekly, and monthly itch scores were calculated. The worst daily itch was defined as the worst of the two daily itch scores (morning and evening) and the mean of these worst itch scores over each of the preceding 7 days was defined as weekly itch score. In the primary analysis of the GLIMMER study, this score was referred to as Mean Worst Daily Itch [34]. The monthly itch score was defined as the worst of the weekly itch scores for that month (4 weeks). Pruritus severity at baseline was categorized by weekly itch score: mild,  ≥ 3− < 4; moderate, ≥ 4 to < 7; and severe, ≥ 7. Patients were required to have an NRS score  ≥ 3 at baseline, so all patients classified as mild had an NRS score of  ≥ 3− < 4. The 5-dimension (5-D) itch scale [36] was also used to assess pruritus through the dimensions of degree, duration, direction, disability, and distribution, measured at the start of the initial study period (Day 1), at baseline (Week 4), Week 12 of treatment, and during the final study period.

The impact of pruritus on sleep was assessed using three different measures. Every morning, patients recorded an NRS score between 0 and 10 (where 0 represents no interference and 10 represents complete interference), evaluating the degree of interference of sleep by pruritus during the preceding night, in their eDiary. The weekly sleep score was defined as the mean of the daily sleep scores over the 7 days preceding each visit, and the monthly sleep score was defined as the worst weekly sleep score for that month (4 weeks). Patients were also asked to answer the following categorical question every morning: “How much did itching interfere with your sleep last night?”, with patients rating their sleep interference due to itch as “not at all”, “a little”, “somewhat”, “quite a bit” or “very much”. Secondly, the PBC-40 questionnaire[37] was used to assess impact on quality of life specific to PBC at each visit, and the impact of pruritus on sleep was assessed using PBC-40 sleep item 8 from the itch domain (“Itching disturbed my sleep”, rated as “never”, “rarely”, “sometimes”, “most of the time”, “always”, or “did not apply/no itch”). Lastly, the impact of pruritus on the sleep item from the disability domain of the 5-D itch scale was used to determine the level of sleep interference. Patients rate the impact of itching on sleep from 1 (never affects sleep) to 5 (delays falling asleep and frequently wakes me up at night) [36]. Out of 147 participants, there were 135 individuals who had both a weekly sleep score and a weekly itch score at Week 16.

### Statistical analyses

Analyses were evaluated in the intent-to-treat (ITT) population, comprising all randomized patients who received at least one dose of study treatment and had a baseline assessment and at least one on-treatment assessment in the main study period. A post-hoc analysis was performed on the ITT population to assess the within participant correlation of change from baseline in monthly itch score versus change from baseline in monthly sleep score at Months 1–3 using the Bland–Altman repeated measures correlation. Further post-hoc correlation analyses were carried out to determine the relationship between itch and sleep using Pearson or Spearman’s correlation. To determine correlation methods, visual inspection was used to assess normality of continuous endpoints. Pearson coefficient was used to calculate correlation between two normal endpoints; Spearman’s rank coefficient was used when at least one endpoint was non-normal. Calculations for confidence intervals (CIs) for both coefficients were performed using the procedure described by Bonett and Wright [38]. Relationship between sleep score and itch response was also assessed post-hoc; a patient was considered an itch responder if they demonstrated an improvement in weekly itch score from baseline of ≥ 2 points, ≥ 3 points, or ≥ 4 points at Week 16. Itch non-responders were patients who achieved less than a 2-, 3-, or 4-point reduction (depending on the threshold) in weekly itch score at Week 16 compared with baseline. Baseline values and changes from baseline in weekly sleep score, 5-D itch and PBC-40 sleep items were summarized using n, mean, range, and 95% CI. Changes from baseline in continuous endpoints by itch responder groups are presented as box plots with mean, median, interquartile range, minimum, maximum, and outliers plotted.

## Results

### Baseline characteristics

At baseline, 76.2% (112/147) of patients from the GLIMMER study had moderate or severe pruritus (Table 1); 40.1% (59/147) of patients reported that itch “somewhat”, “quite a bit”, or “very much” interfered with their sleep based on the categorical itch question. Post-hoc analysis revealed that sleep, as assessed by weekly sleep score, was significantly worse in patients categorized with severe pruritus (weekly sleep score [95% CI]: 7.36 [6.92; 7.81]) compared with moderate (3.22 [2.83; 3.60]) and mild (1.85 [1.53; 2.17]) categories with non-overlapping CIs (Table 1). Similar findings were found for impact of pruritus on sleep measured by 5-D itch sleep item score and PBC-40 item 8 (“Itching disturbed my sleep”) (Table 1).

**Table 1:** Patient clinico-demographics and sleep scores by baseline pruritus severity for all patients in GLIMMER (N = 147)^†^

Parameter	Mild pruritus severity^‡^ n = 35	Moderate pruritus severity^‡^ n = 76	Severe pruritus severity^‡^ n = 36
Age, years, median (range)	61.0 (38–78)	57.5 (28–78)	49.5 (31–71)
Female, n (%)	32 (91.4)	72 (94.7)	34 (94.4)
ALP, IU/L, median (range)	133.0 (46–505)	126.0 (47–1443)	217.0 (66–987)
PBC disease stage, n (%)^§^			
Stage 1	9 (25.7)	17 (22.4)	12 (33.3)
Stage 2	7 (20.0)	17 (22.4)	11 (30.6)
Stage 3	5 (14.3)	13 (17.1)	5 (13.9)
Stage 4	3 (8.6)	2 (2.6)	1 (2.8)
Mean baseline weekly sleep score (95% CI)^¶^	1.85 (1.53; 2.17)	3.22 (2.83; 3.60)	7.36 (6.92; 7.81)
Mean baseline 5-D impact on sleep (95% CI)^††^	2.97 (2.50; 3.45)	2.80 (2.50; 3.11)	4.49 (4.20; 4.77)
Mean baseline PBC-40 itch sleep disturbance (95% CI)^‡‡^	2.86 (2.57; 3.15)	2.92 (2.68; 3.16)	4.36 (4.07; 4.65)

^†^Baseline values for weekly sleep score, 5-D itch, and PBC-40 sleep items were summarized using n, mean, range and 95% CI

^‡^Pruritus severity was categorized by absolute weekly itch score: mild,  ≥ 3− < 4; moderate, ≥ 4 and < 7; and severe, ≥ 7

^§^Stage 1 defined as florid duct lesion or chronic non-suppurative destructive cholangitis; Stage 2 defined as proliferation of the small bile ductules; Stage 3 defined as fibrosis or scarring present; Stage 4 defined as presence of cirrhosis [20]. Not all patients had staging data available; percentages for each severity category do not sum to 100% due to missing data

^¶^NRS range 0–10

^††^Derived from the sleep item within the disability domain. Score range 1–5 [36]

^‡‡^Derived from responses to the statement ‘Itching disturbed my sleep’. Score range 1–5 [37]

*ALP* alkaline phosphatase; *CI* confidence interval; *NRS* numerical rating scale; *PBC-40* quality of life measure for primary biliary cholangitis; *5-D* 5-dimension

### Relationship between pruritus and sleep

**Fig. 1:**
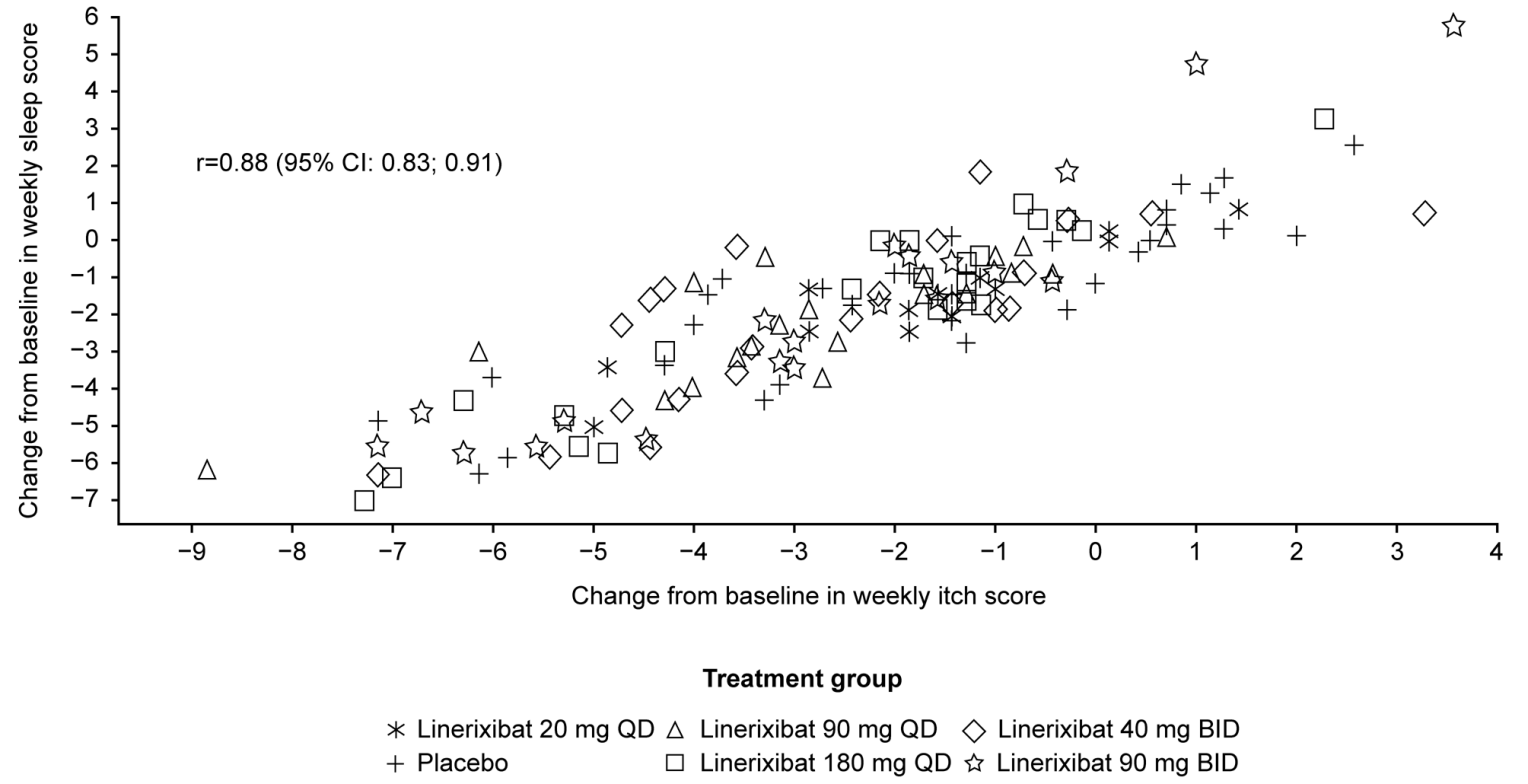
Correlation between change from baseline in weekly sleep and itch scores at Week 16. n = 135 patients. Pearson product-moment correlation was used. *BID* twice daily, *CI* confidence interval, *QD* once daily

**Fig. 2:**
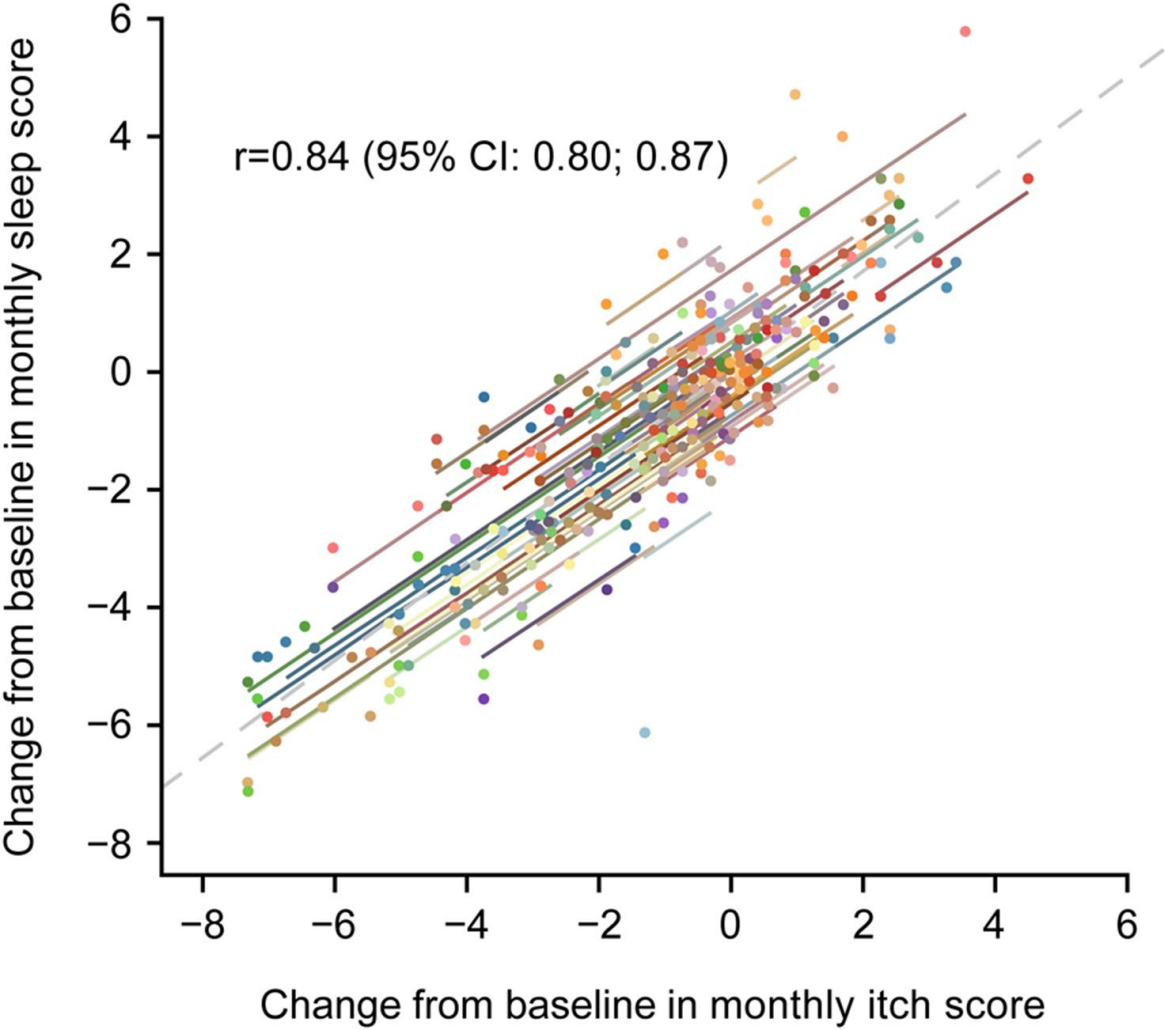
Correlation of CFB in monthly sleep and itch scores at Week 16. N = 147 patients (intent-to-treat population). Bland–Altman repeated measures correlation. Each colored dot and corresponding line is associated with an individual patient. *CFB* change from baseline, *CI* confidence interval

To evaluate the relationship between pruritus and sleep, this post-hoc analysis was conducted utilizing the GLIMMER study data to assess whether improvements in pruritus correlated with improvements in sleep. At Week 16, a strong correlation was observed between change from baseline in weekly sleep score and change from baseline in weekly itch score (r = 0.88 [95% CI: 0.83; 0.91]) (Fig. 1). Similar correlations were observed between change from baseline in monthly sleep score and monthly itch score (Fig. S1). Post-hoc analysis using the Bland–Altman statistical method showed a correlation between change from baseline in monthly itch score and change from baseline in monthly sleep score (Months 1–3) (r = 0.84 [95% CI: 0.80; 0.87]) (Fig. 2), indicating that the worsening of pruritus may contribute to increased sleep interference, and that the improvement of pruritus may contribute to decreased sleep interference.

### Relationship between improvement in pruritus and sleep

**Fig. 3:**
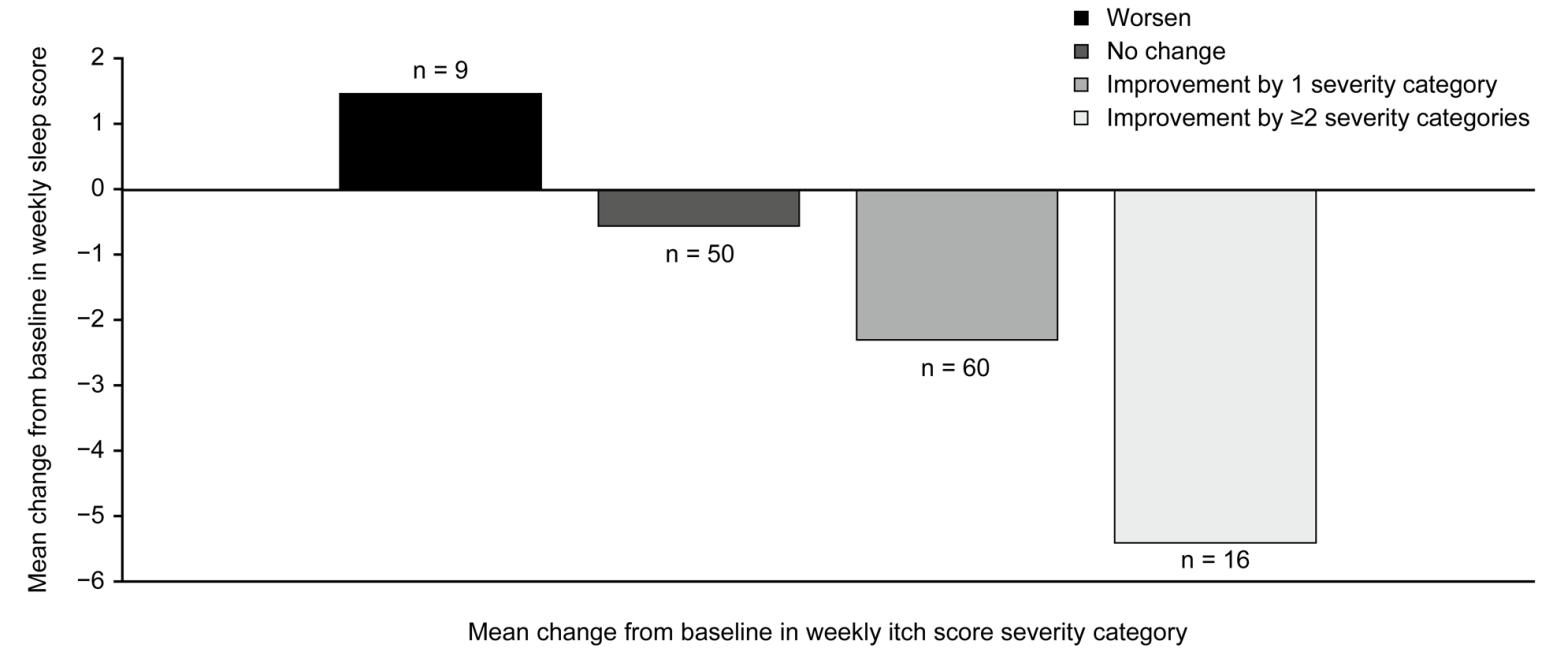
Reduction in sleep interference by pruritus severity improvements from baseline at Week 16. n = 135 patients. Improvement by two severity categories corresponds to change in pruritus from severe to mild or moderate to none

The relationship between improvement in pruritus and improvement in sleep was examined post-hoc by assessing whether reported improvements in pruritus severity categories (i.e., mild, moderate, severe) were associated with reduced sleep interference due to pruritus. Patients who experienced improvements in weekly itch score severity category from baseline at Week 16 also demonstrated improved weekly sleep score at Week 16 (Fig. 3). The extent of improvement in pruritus over the course of the study also seemed to have an impact, as patients who improved by two or more pruritus severity categories from baseline (i.e., severe to mild; moderate to none) reported greater improvements in sleep interference scores at Week 16 compared with those who only improved by one category.

**Fig. 4:**
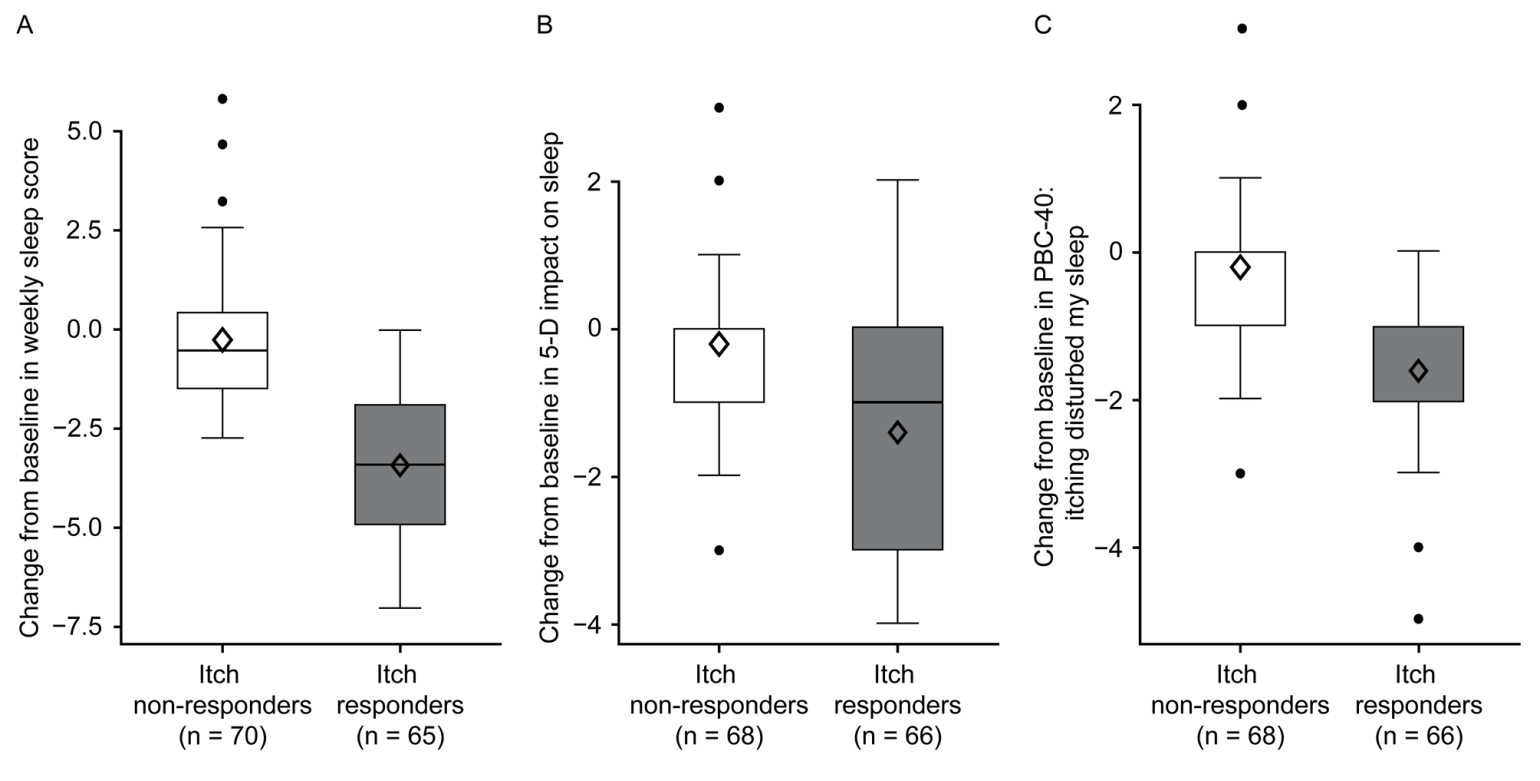
Itch responders analysis: CFB in weekly sleep score (n = 135), 5-D itch (n = 134), and PBC-40 itch domain sleep item (n = 134). **A** weekly sleep score; **B** 5-D itch scale impact of itch on sleep (disability domain sleep item) and **C** PBC-40 itch domain sleep item 8, impact of itch on sleep. A patient was considered an itch responder if they had a weekly itch score improvement from baseline of at least 2 points on the itch NRS at Week 16. Changes from baseline in continuous endpoints by itch responder groups are presented as box plots with mean (diamond), median, interquartile range, minimum, maximum, and outliers plotted. *CFB* change from baseline, *PBC-40* quality of life measure for primary biliary cholangitis, *5-D* 5-dimension

To further characterize the relationship between improvements in pruritus and sleep, weekly sleep score was assessed post-hoc in patients with improvements in pruritus (i.e., itch responders). Patients were considered itch responders if they exhibited weekly itch score improvement of ≥2 points from baseline at Week 16. Patients who demonstrated itch response experienced greater improvements in weekly sleep score, in the sleep items from the 5-D itch scale and in the PBC-40 sleep measure, compared with itch non-responders (Fig. 4). Exploring different thresholds to define itch responders, improvements from baseline of ≥3 or ≥4 points showed consistent and greater improvements in weekly sleep score, as well as in the sleep items from 5-D itch and PBC-40 measures, compared with itch non-responders (Fig. S2). Similar findings were observed with monthly sleep score (Fig. S3).

## Discussion

Pruritus and fatigue are two of the most common and debilitating conditions impacting patients with PBC. To reduce the negative impact of these conditions on patients and their quality of life, it is essential to understand the relationship between pruritus and sleep and to determine whether improvements in pruritus have the potential to result in improved sleep. Although several studies have investigated the association between pruritus and sleep [11, 12, 39–42], this relationship has not previously been well characterized in patients with PBC. This post-hoc analysis of GLIMMER was the first and largest trial to extensively explore the directional relationship between pruritus and sleep in PBC in the entire population, regardless of treatment, using robust methodology [34]. The findings from this analysis demonstrate that there is a clear correlation between pruritus severity and the impact of pruritus on sleep interference in patients with PBC. Sleep interference was worse in patients with moderate or severe pruritus compared with those with mild pruritus, with a strong correlation between changes from baseline in weekly itch score and weekly sleep score at Week 16. Patients who reported an improvement in pruritus severity category from baseline to Week 16 exhibited improvements in sleep as measured by weekly sleep score. Further, mean improvement in weekly sleep score was greater in those with more substantial improvements in pruritus. Itch responders on average showed improved sleep compared with itch non-responders, which was confirmed by multiple different measures (weekly sleep score, reduced 5-D itch impact on sleep scores, and decreased PBC-40 sleep disturbance scores) across a range of itch response thresholds. Indeed, the reduction in pruritus-related sleep interference in patients with a ≥ 2-category improvement in pruritus was more than twice that in those with a 1-category improvement. Thus, improvement in pruritus is likely to lead to a concomitant reduction in sleep interference in patients with PBC.

In GLIMMER, daily sleep scores improved in all groups, including placebo, and there was a high concordance between improvements in itch and sleep scores [34]. The high placebo response is not unusual in pruritus studies [28, 43, 44], as well as studies that rely on subjective patient-reported outcomes [45]. GLIMMER was a dose-ranging study; changes in sleep score over the treatment period were more notable for the BID dose groups, concordant with significant improvements in pruritus. As such, this analysis was performed to further explore the relationship between pruritus severity and sleep-interference due to pruritus in patients with PBC, regardless of treatment group. The Phase 3 GLISTEN trial will further examine the effect of an optimized dose of linerixibat (40 mg BID) on sleep interference due to pruritus.

This post-hoc analysis of the GLIMMER study builds on results from a Phase 2a randomized, double-blind trial of 22 patients with PBC and pruritus, which demonstrated a significant improvement in pruritus and sleep NRS with 2 weeks of linerixibat treatment compared with placebo [24]. Two small open-label studies have shown improvements in pruritus-related sleep disturbance in patients with PBC; however, neither included a placebo comparator. The first was a Phase 2b study where 4 out of 10 patients treated with odevixibat reported improvements in pruritus as assessed by the PBC-40 itch domain and no longer experienced sleep disturbance due to pruritus [29]. The second was a Phase 2 open-label study of seladelpar in 101 patients with PBC where improvements in pruritus-related sleep disturbance, measured using the PBC-40 sleep item, were reported after 1 year [46]. Two earlier studies did include a placebo arm, including an analysis on the efficacy of sertraline in patients with cholestatic pruritus [47]. Fifty-seven percent of patients had PBC, and while sleep disturbance due to pruritus was reduced with open-label sertraline, improvements were similar in the sertraline and placebo arm when patients received double-blind treatment. The second study assessed the antipruritic effect of naltrexone in 16 patients with PBC or primary sclerosing cholangitis. Patients treated with naltrexone experienced reduced daytime and nighttime pruritus which correlated with reduced sleep disturbance [48]. While it is unclear whether improvements were associated with the specific study drug or a placebo effect, a commonality in each of these studies was that the improvement in pruritus resulted in an improvement in sleep in patients with chronic liver disease.

Despite the differences in study design and the variety of measures used to assess pruritus and sleep, there is arguably a suggestion in the published literature of an association between pruritus and sleep interference in PBC, as seen in other conditions [39, 40]. Although this post-hoc analysis demonstrates the potential clinical utility of PRO measures of sleep, the patient-reported nature of these measures may lead to a level of self-report bias. Other methods, such as actigraphy technology, monitor rest/activity cycles during sleep, may allow for an objective daily measure of sleep and sleep disturbance. Actigraphy was initially utilized in the GLIMMER trial as an optional component of the study but was discontinued due to negative patient feedback on the wearable device. The continued advancement in tools that can accurately and objectively assess sleep may provide more effective and practical means of measuring sleep in future studies [49]. Additionally, these analyses used simple correlations to assess the strength of the relationship between itch and sleep. These relationships could be further explored using modeling to adjust for covariates.

Lastly, one limitation of the current post-hoc analysis was the use of a single-item NRS for assessing sleep in GLIMMER, as it only evaluated sleep interference and was not able to differentiate which aspects of sleep were affected, such as quantity, quality, and effects on daily activities. In addition, since the protocol permitted patients with itch NRS ≥ 3 at baseline (Week 4) to be randomized, approximately 25% of the population with mild pruritus entered the treatment period [34]. Thus, the ability to fully detect an impact on pruritus, and any corresponding improvement in sleep in such patients, may have been limited. However, inclusion of patients with a wider range of pruritus is expected to have minimized the confounding effect of pruritus severity.

## Conclusions

In conclusion, this post-hoc analysis of GLIMMER demonstrates a strong correlation between pruritus and sleep in patients with PBC, with the presence of worse pruritus correlating with worse sleep. Furthermore, reduction in PBC pruritus led to improved sleep, with greater improvements in pruritus associated with greater improvements in sleep. This is likely to have a beneficial impact on quality of life in patients with PBC. Future larger studies, such as the ongoing Phase 3 GLISTEN trial, will aim to better understand the effect of linerixibat on pruritus and sleep.

### Electronic supplementary material

Below is the link to the electronic supplementary material.

Supplementary Material 1

## Data Availability

Anonymized individual participant data and study documents can be requested for further research from https://www.gsk-studyregister.com/en/.

## Abbreviations

5-D: 5-dimension
ALP: Alkaline phosphatase
BID: Twice daily
CI: Confidence interval
eDiary: Electronic diary
FDA: Food and Drug Administration
IBAT: Ileal bile acid transporter
ITT: Intent-to-treat
NRS: Numerical rating scale
PBC: Primary biliary cholangitis
QD: Once daily
UDCA: Ursodeoxycholic acid

## Declarations

### Clinical trial registration

clinicaltrials.gov, NCT02966834. Registered 15 November 2016.
