# Supplementary material for: Relationship between pruritus and sleep in participants with primary biliary cholangitis in the Phase 2b GLIMMER trial
Source: J Patient Rep Outcomes. 2024 Jun 12;8:60. doi: 10.1186/s41687-024-00722-y (PMC11166618; doi:10.1186/s41687-024-00722-y)
Supplement: Supplementary file 1 — Supplementary Material 1 [file 41687_2024_722_MOESM1_ESM.docx]

**Relationship between pruritus and sleep in participants with primary biliary cholangitis in the Phase 2b GLIMMER trial: Supplementary material**

#

# Table of Contents

[Fig. S1. Scatter plot illustrating the correlation^†^ between change from baseline in sleep (monthly sleep score) and itch (monthly itch score) at Week 16. 2](#_Toc134009489)

[Fig. S2. Change from baseline in: A) weekly sleep score; B) 5-D itch scale disability domain sleep item; and C) PBC-40 itch domain sleep item 8, ‘itching disturbed my sleep’, for itch responders versus non-responders^†^ 3](#_Toc134009490)

[Fig. S3. Change from baseline in monthly sleep score in itch responders versus non-responders at Week 16 5](#_Toc134009491)

## Fig. S1. Scatter plot illustrating the correlation^†^ between change from baseline in sleep (monthly sleep score) and itch (monthly itch score) at Week 16.


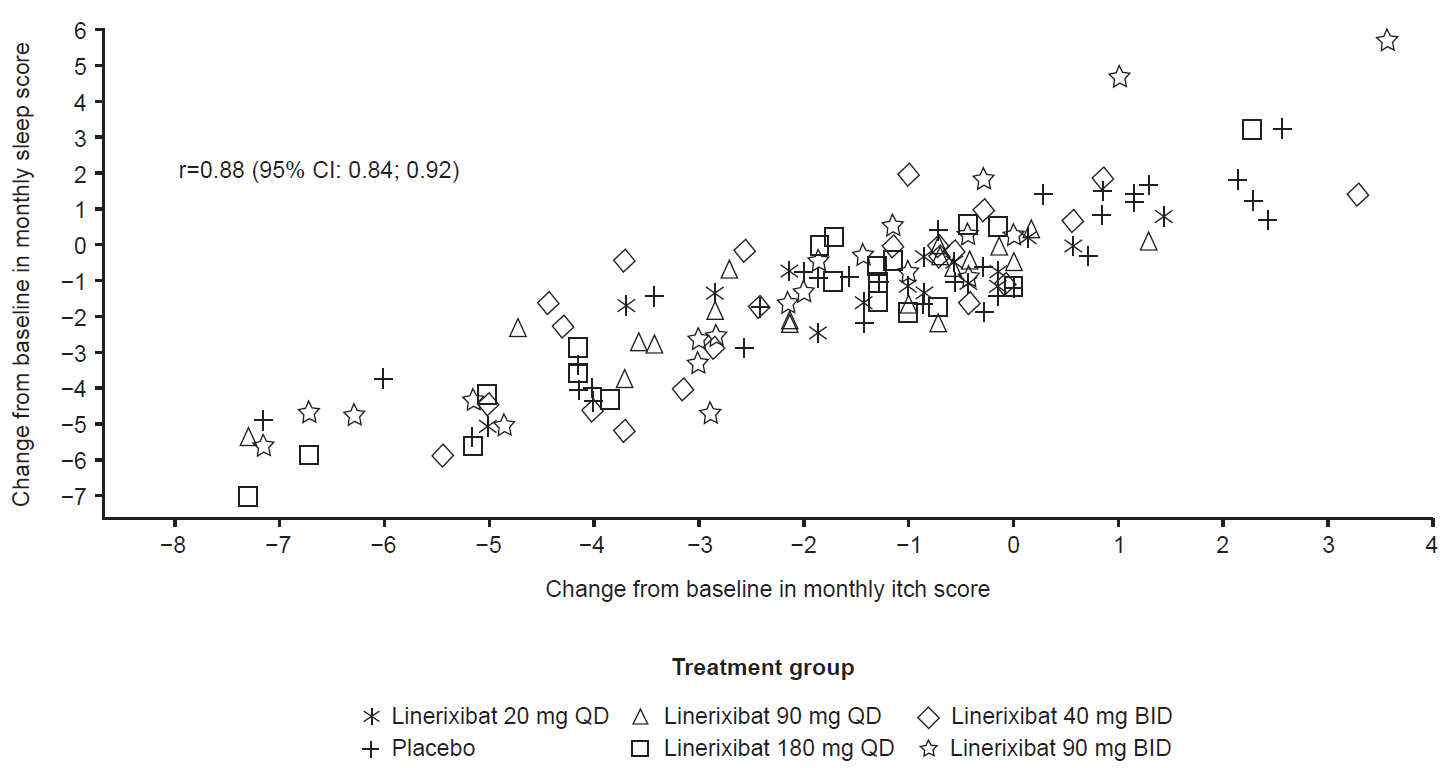


n=135 patients. ^†^Pearson product-moment correlation was used.

BID, twice daily; CI, confidence interval; QD, once daily.

## Fig. S2. Change from baseline in: (A) weekly sleep score (n=135); (B) 5-D itch scale disability domain sleep item (n=134); and (C) PBC-40 itch domain sleep item 8, ‘itching disturbed my sleep’, for itch responders versus non-responders (n=134).^†^


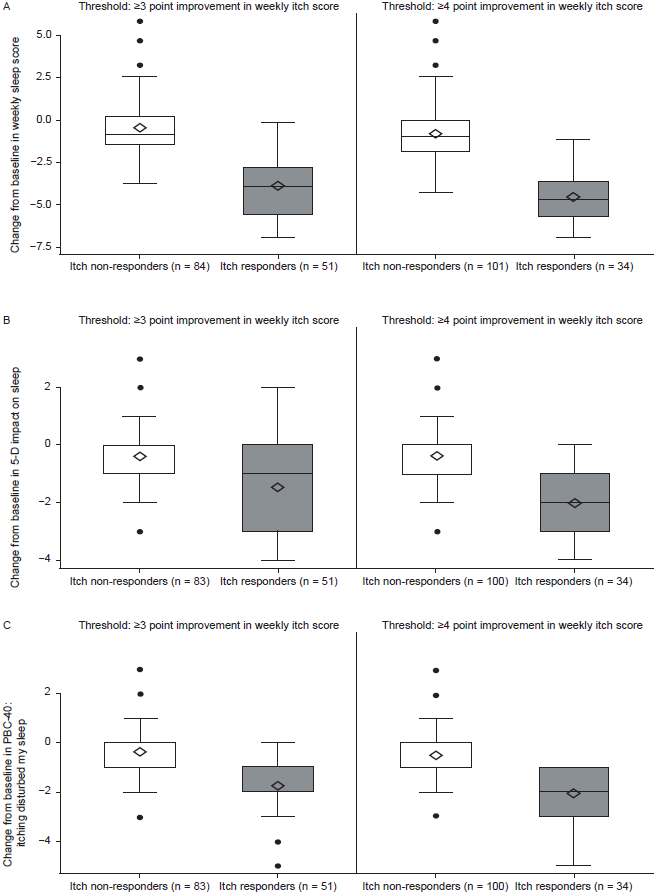


A patient was considered an itch responder if they had a weekly itch score improvement from baseline of at least 3, or 4 points, respectively, at Week 16. Changes from baseline in continuous endpoints by itch responder groups are presented as box plots with mean (Diamond), median, interquartile range, minimum, maximum, and outliers plotted.

^†^Data for patients with at least a 2-point improvement in weekly itch score are shown in Figure 4.

PBC-40, quality of life measure for primary biliary cholangitis; 5-D, 5-dimension itch scale.

## Fig. S3. Change from baseline in monthly sleep score in itch responders versus non-responders at Week 16.


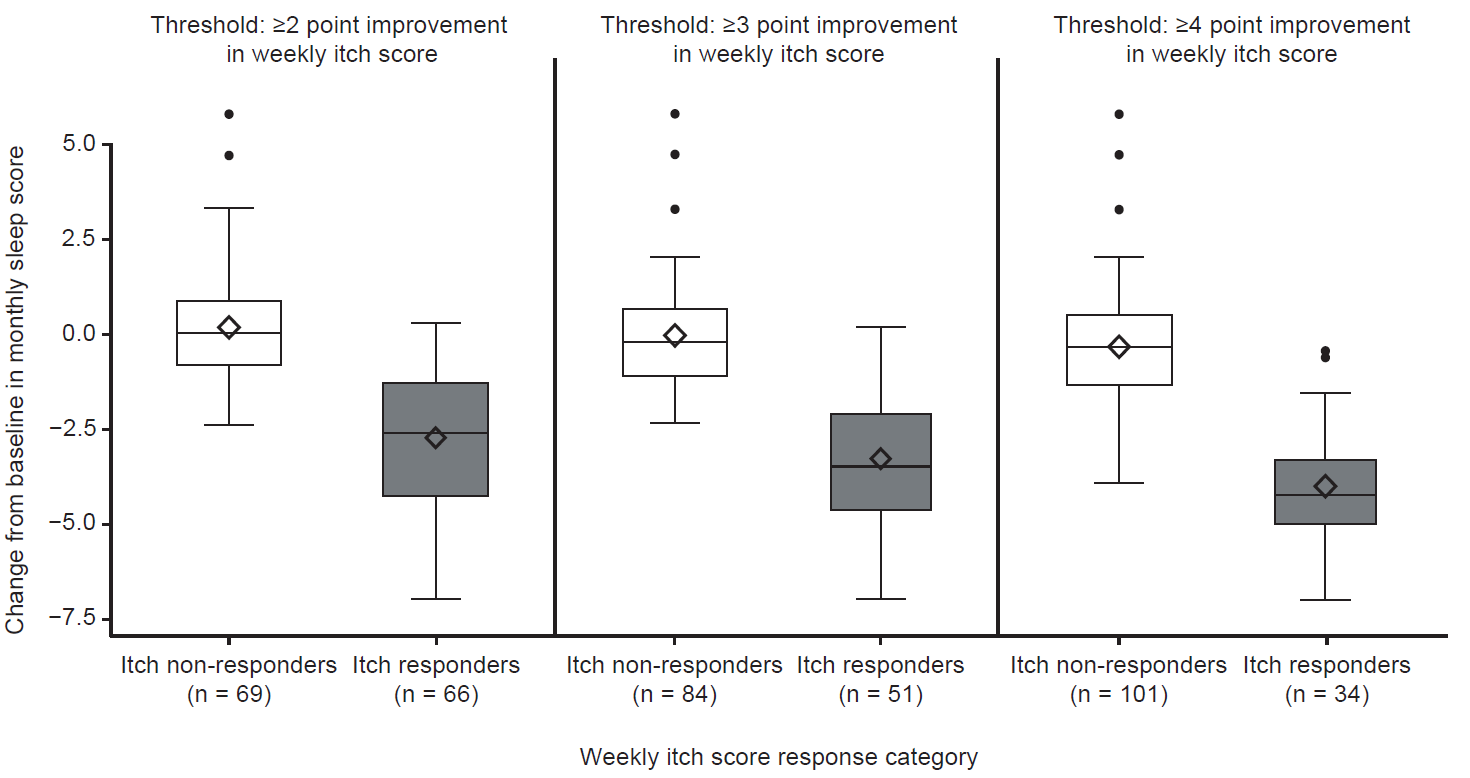


n=135 patients. A patient was considered an itch responder according to three different thresholds if they had a weekly itch score improvement from baseline of at least 2, 3, or 4 points, respectively, at Week 16. Changes from baseline in continuous endpoints by itch responder groups are presented as box plots with mean (Diamond), median, interquartile range, minimum, maximum, and outliers plotted.
